# Reprogramming the endogenous type I CRISPR‐Cas system for simultaneous gene regulation and editing in *Haloarcula hispanica*

**DOI:** 10.1002/mlf2.12010

**Published:** 2022-03-17

**Authors:** Kaixin Du, Luyao Gong, Ming Li, Haiying Yu, Hua Xiang

**Affiliations:** ^1^ State Key Laboratory of Microbial Resources, Institute of Microbiology Chinese Academy of Sciences Beijing China; ^2^ University of Chinese Academy of Sciences Beijing China; ^3^ CAS Key Laboratory of Microbial Physiological and Metabolic Engineering, Institute of Microbiology Chinese Academy of Sciences Beijing China

**Keywords:** CRISPR‐Cas system, gene editing, gene regulation, type I

## Abstract

The type I system is the most widely distributed CRISPR‐Cas system identified so far. Recently, we have revealed the natural reprogramming of the type I CRISPR effector for gene regulation with a crRNA‐resembling RNA in halophilic archaea. Here, we conducted a comprehensive study of the impact of redesigned crRNAs with different spacer lengths on gene regulation with the native type I‐B CRISPR system in *Haloarcula hispanica*. When the spacer targeting the chromosomal gene was shortened from 36 to 28 bp, transformation efficiencies of the spacer‐encoding plasmids were improved by over three orders of magnitude, indicating a significant loss of interference. However, by conducting whole‐genome sequencing and measuring the growth curves of the hosts, we still detected DNA cleavage and its influence on cell growth. Intriguingly, when the spacer was shortened to 24 bp, the transcription of the target gene was downregulated to 10.80%, while both interference and primed adaptation disappeared. By modifying the lengths of the spacers, the expression of the target gene could be suppressed to varying degrees. Significantly, by designing crRNAs with different spacer lengths and targeting different genes, we achieved simultaneous gene editing (*cdc6E*) and gene regulation (*crtB*) for the first time with the endogenous type I CRISPR‐Cas system.

## Impact statement

This is the first systematic study of the effects of spacer length on different CRISPR functionalities, including target interference, primed adaptation, and transcription inhibition. As the type I system is widespread among prokaryotes, this study has provided a novel and significant strategy in a wide‐range of archaea and bacteria with endogenous CRISPR‐Cas systems, for delicate gene regulation or editing, just by modulating the crRNA length for the target gene.

## INTRODUCTION

The CRISPR‐Cas system is an adaptive immune system that is widely distributed among archaea and bacteria^1^, ^2^, ^3^. Its basic immune mechanisms include three processes: adaptation, crRNA biogenesis, and interference^4^. According to the latest classification, CRISPR‐Cas systems are divided into 2 classes, 6 types, and more than 30 subtypes^3^. Class 1, which includes type I, type III, and type IV systems, is characterized by the effector participating in interference as a complex of several Cas proteins. Contrastingly, class 2, which includes type II, type V, and type VI systems, is characterized by the effector being a single protein^3^, ^5^, ^6^, ^7^.

The type I system is the most widely distributed CRISPR system in sequenced genomes^8^. The effector complex of the type I system is called the CRISPR‐associated complex for antiviral defense (Cascade). There are two types of adaptation mechanisms: naïve adaptation and primed adaptation^9^, ^10^, ^11^. Naïve adaptation generally requires the participation of Cas1 and Cas2, and results in low efficiency and autoimmunity^9^, ^12^. Primed adaptation is mainly reported in type I systems^13^ and generally requires the participation of Cas1, Cas2, Cascade effector, Cas3, and a pre‐existing spacer partially matching the target sequence^10^, ^11^, ^14^, ^15^. During primed adaptation, new spacers can be efficiently obtained around the target sequence of the priming crRNA^10^, ^11^, ^15^, ^16^, ^17^.

In previous studies, we analyzed the different requirements between primed adaptation and interference of the *Haloarcula hispanica* type I‐B CRISPR system^18^. Our findings show that primed adaptation can tolerate more structural and size variations of the crRNA than interference. Besides, some studies show that, by reprogramming the spacer length of crRNAs, Cascade effectors can be formed stably with altered stoichiometry^19^, ^20^, ^21^. Significantly, we recently discovered the inherent gene regulation function of the type I‐B CRISPR system in which the crRNA‐resembling antitoxin (i.e., CreA) RNA served as a guide for the Cascade to inhibit the Cascade‐repressed toxin (i.e., CreT) transcription by matching an 11 bp seed region^22^. Meanwhile, both primed adaptation and interference disappeared. These findings suggest that the spacer length can be modified to affect functionalities of the CRISPR system, including interference, primed adaptation, and gene regulation.

Simultaneous gene editing and regulation can be achieved by utilizing a single active effector in class 2 systems via altering the spacer length^23^, ^24^. However, whether this is feasible in class 1 systems remains unclear. Herein, we conducted the first systematic study of different CRISPR functionalities of the I‐B CRISPR system, including interference, primed adaptation, and gene inhibition. Based on it, using the active Cas3, the Cascade effector, and redesigned crRNAs with different spacer lengths, we achieved gene editing and regulation simultaneously for the first time with the endogenous type I CRISPR‐Cas system. The findings of this study can help researchers better understand the flexibility of the widespread type I system, and provide a novel and a more general strategy for delicate gene editing and regulation in a wide range of archaea and bacteria with endogenous CRISPR‐Cas systems, especially for those environmental or industrial strains and clinic pathogens without efficient genetic tools.

## RESULTS

### Interference effect is significantly reduced when the spacer is shortened to 30 bp or less

**Figure 1:**
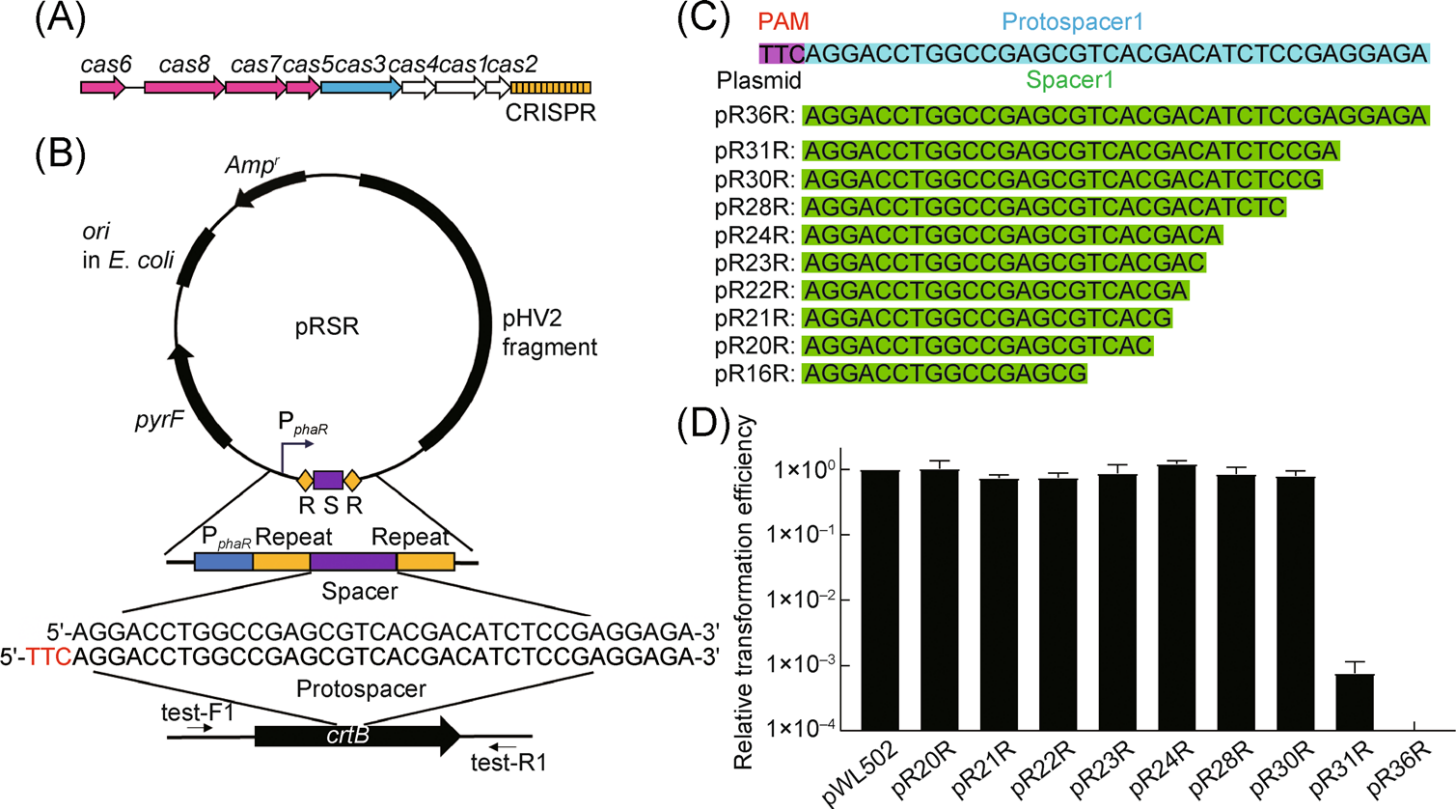
Transformation efficiencies of DF60 are improved with pRSR plasmids when spacers are shortened to 30 bp or less. (A) Schematic of the type I‐B CRISPR‐Cas system in *Haloarcula hispanica* DF60. (B) Schematic of pRSR plasmid carrying a mini‐CRISPR with a spacer targeting the *crtB* gene. TTC in red font represents the PAM sequence. (C) Schematic of spacers with different lengths contained in pRSR plasmids. Magenta background, the PAM sequence; blue background, the protospacer sequence; green background, the spacer sequences. (D) Relative transformation efficiencies of DF60 with pRSR plasmids carrying spacers of different lengths (20–36 bp), compared to that with the control plasmid pWL502. Error bars indicate the standard deviation (SD) of three independent replicates.

The type I‐B CRISPR‐Cas system in *H. hispanica* is composed of a CRISPR array and eight *cas* genes (Figure 1A). This system can resist the invasion of foreign nucleic acids (viruses or plasmids) and reduce plaque‐forming units (PFUs) by six to seven orders of magnitude^15^ or colony‐forming units (CFUs) by three orders of magnitude^25^, ^26^.

To systematically study the impact of spacer length on the functional processes of the type I‐B CRISPR‐Cas system in the *H. Hispanica* DF60 strain, a series of plasmids (based on pWL502) with mini‐CRISPR sequences targeting the chromosomal *crtB* (HAH_RS12450) gene were constructed (Table S1). The *crtB* gene encodes a phytoene synthase, and the disruption or knockout of this gene causes the color of *H. hispanica* cells to change from red to white^26^, ^27^. The target site was located in the middle of the template strand with a 5ʹ‐TTC‐3ʹ PAM sequence (Figure 1B). Considering that the size of newly acquired spacers during adaptation varies from 32 to 39 bp, with 36 bp being the most prevalent^16^, we truncated the *crtB*‐targeting spacer from the 3ʹ end to different lengths (20, 21, 22, 23, 24, 28, 30, and 31 bp) based on the 36 bp spacer (Figure 1C)^26^. Then we cloned the mini‐CRISPR sequences with spacers of different lengths into the vector pWL502 carrying the selective marker gene, *pyrF*, to construct expression plasmids pRSR (S indicates the different lengths of spacers), which were then transferred into DF60 cells. Notably, the transformation efficiencies of plasmids with spacers of 31 and 36 bp were reduced by about three orders of magnitude, compared to that of the control plasmid pWL502 (Figure 1D), which indicates that the CRISPR‐Cas system has a powerful interference function. Importantly, when spacers were shortened to 30 bp or less, the transformation efficiencies of plasmids were almost equivalent to that of pWL502 (Figure 1D), indicating a significant loss of interference with the shortened crRNAs, which was in agreement with our previous results targeting the HHPV‐2 virus^18^.

### Primed adaptation disappears when the spacer is shortened to 24 bp or less

**Figure 2:**
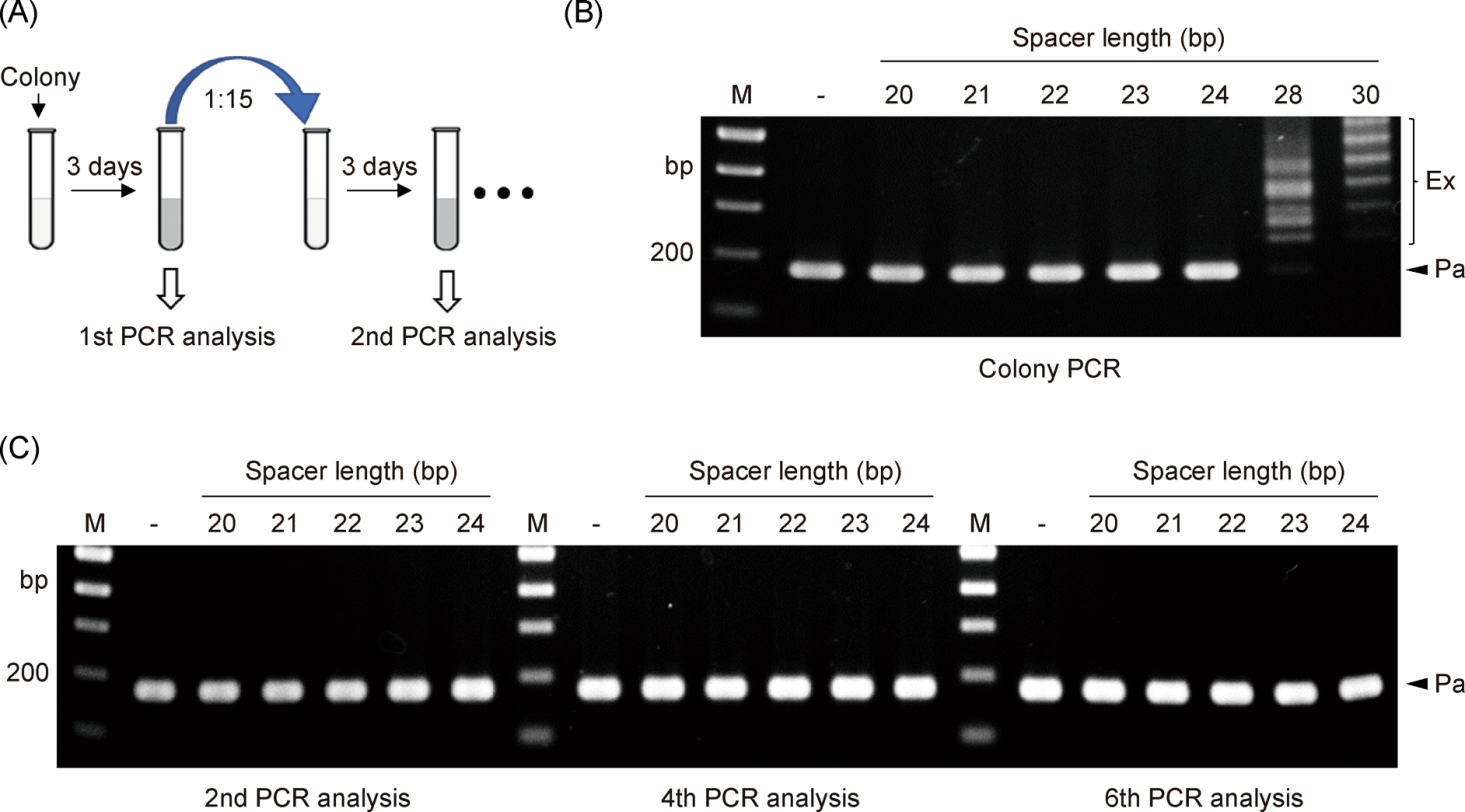
CRISPR‐Cas system can not acquire new spacers via primed adaptation when spacers are shortened to 24 bp or less. (A) The experimental procedure to monitor the adaptation primed by shortened spacers in DF60 cells. (B) Colony polymerase chain reaction (PCR) analyses of primed adaptation in transformants with shortened spacers. (C) PCR analyses of primed adaptation in the second, fourth, and sixth cultures of transformants with shortened spacers. M, dsDNA size marker; –, the empty pWL502 control; Pa, parental bands; Ex, expanded bands.

To assess the impact of shortened spacers on the primed adaptation of the CRISPR‐Cas system, transformants with plasmids containing different shortened spacers targeting *crtB* were subjected to colony polymerase chain reaction (PCR) analysis with primers for detecting adaptation (Figure 2A). The primer Leader‐F and Spacer1‐R (Table S2) were located in the leader and the first spacer of the CRISPR array in *H. hispanica* chromosome, respectively. For transformants with spacer lengths of 28 and 30 bp, there was a gradient distribution of bands longer than the parental band (Figure 2B), indicating the acquisition of new spacers resulting from efficient primed adaptation. Notably, when we inoculated the above transformants into the liquid medium, it was hard for the cells with spacer lengths of 28 and 30 bp to grow. We speculated that primed adaptation and interference resulting from acquired new spacers might cause cleavage to the chromosome and impair cell growth. When the spacer length was between 20 and 24 bp, there were only the parental bands observed for the transformants (Figure 2B), depicting that no new spacers were acquired. Further, when the transformants were inoculated into the liquid medium and subcultured every 3 days for five times continuously (Figure 2A), cells grew well and primed adaptation was still not observed, even in the sixth subculture (Figure 2C).

### The expression of target gene is repressed at different levels when the spacer length varies

**Figure 3:**
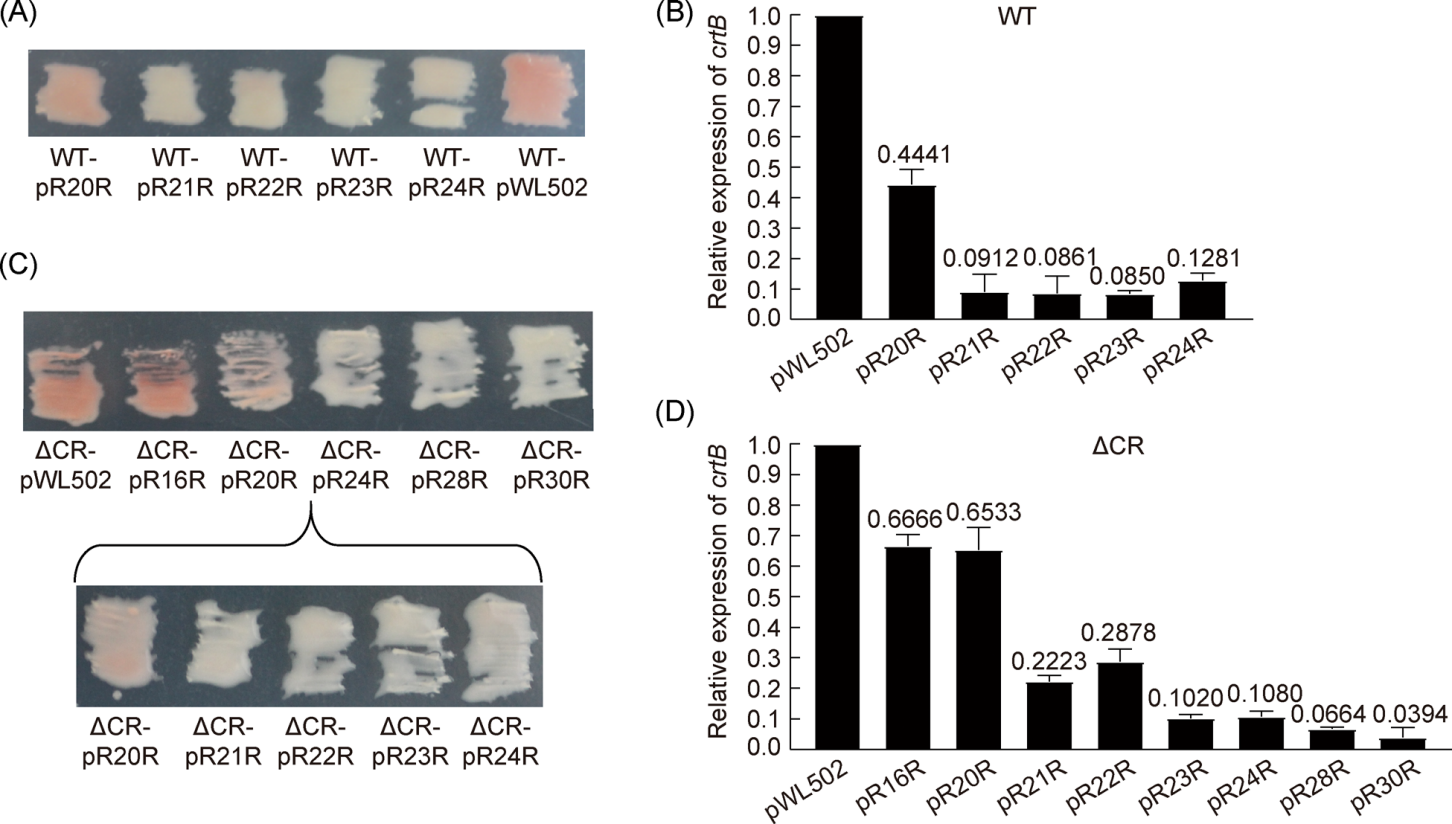
Spacers of different lengths can repress the *crtB* gene expression at different levels. (A) *Haloarcula hispanica* DF60 (WT) transformants with pRSR carrying different spacers displayed different colors. (B) Relative expression level of the *crtB* gene in *H. hispanica* DF60 (WT) transformants with pRSR carrying spacers of different lengths compared to the control plasmid pWL502. (C) ΔCR transformants with pRSR carrying different spacers displayed different colors. (D) Relative expression level of the *crtB* gene in *H. hispanica* ΔCR transformants with pRSR carrying spacers of different lengths compared to the control plasmid pWL502. Error bars indicate the standard deviation (SD) of three independent replicates.

As knockout of *crtB* gene leads to the color change of *H. hispanica* cells from red to white^27^, we further investigated the inhibition of the *crtB* expression and the color change by *crtB*‐targeting spacers in a mini‐CRISPR. Transformants with pR20R were pink, while transformants with plasmids carrying spacers of 21–24 bp were white (Figure 3A). The colors of transformants with shortened spacers were lighter than that of the pWL502 transformants to varying degrees, indicating that the expression of the *crtB* gene was repressed at different levels. Upon quantitatively measuring the relative transcriptional level of the *crtB* gene in different transformants using quantitative reverse transcription‐PCR (qRT‐PCR) with the primers crtBQF1/crtBQR1, we found that the amount of *crtB* mRNA in the transformants with spacer lengths of 20, 21, 22, 23, and 24 bp were reduced to 44.41%, 9.12%, 8.61%, 8.50%, and 12.81% of that in control transformants, respectively (Figure 3B). The gene repression effects with spacer lengths of 21–24 bp were much stronger than that with 20 bp spacer, which was consistent with the color of different transformants.

### Spacer length impacts target gene expression in strains with different CRISPR backgrounds

As there are 13 spacers in the CRISPR array of the DF60 strain, the endogenous crRNAs may influence the regulation of target genes through competitive binding with Cascade. To avoid this influence, we used the ΔCR strain, constructed by knocking out the CRISPR array and leader sequence in DF60^28^, as the host strain. The CRISPR‐Cas system could not perform primed adaptation in the ΔCR strain. Plasmids carrying spacers of different lengths and pWL502 were transferred into ΔCR cells. When spacers were longer than 20 bp, the color of the corresponding transformants was white (Figure 3C), indicating strong repression of *crtB* gene expression. The relative transcript levels of the *crtB* gene in different transformants were measured via qRT‐PCR (Figure 3D). When the spacer lengths were 16, 20, 21, 22, 23, 24, 28, and 30 bp, the amount of *crtB* mRNA in the transformants were reduced to 66.66%, 65.33%, 22.23%, 28.78%, 10.20%, 10.80%, 6.64%, and 3.94% of that in the control, respectively. Consistent with the results in DF60, when the spacer length was 20 bp, the effect of gene repression was significantly reduced compared to that with a spacer length of 21 bp. Unexpectedly, the gene repression effects of *crtB* in DF60 were close to, sometimes better than, those in ΔCR. We assumed that the expression of Cas proteins was so strong that Cascade could combine with crRNAs from both native spacers and mini‐CRISPR spacers.

In previous studies of gene regulation using the type I CRISPR‐Cas system, the *cas3* gene was knocked out to prevent the CRISPR‐Cas system from cleaving target DNA^19^, ^29^, ^30^, ^31^. To compare the repression function with and without *cas3*, plasmids with spacers of 20, 24, 28, and 30 bp were transferred into DC3 strain in which the *cas3* gene was knocked out^15^. The transcriptional levels of the *crtB* gene were 69.21%, 10.81%, 13.24%, and 4.76% of that in the control, respectively (Figure S1). The gene repression effects of *crtB* in ΔCR were close to, sometimes better than, those in DC3. These results indicate that when using the type I CRISPR‐Cas system for gene regulation, effective repression of the target gene can be achieved with or without the *cas3* gene.

### Twenty‐four base pairs or shorter can be secure spacer lengths for gene repression

**Figure 4:**
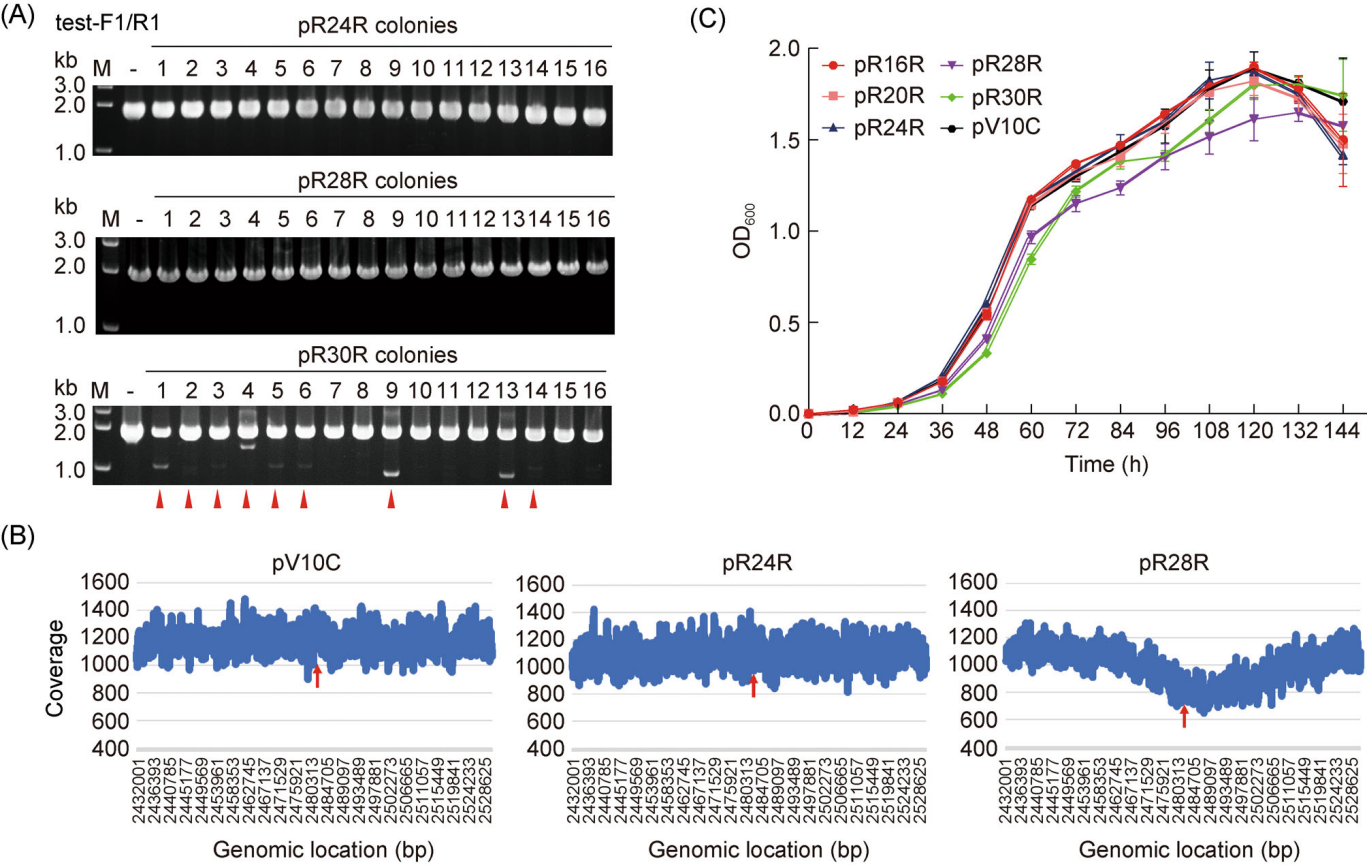
Twenty‐four base pairs or shorter can be the length of a spacer for secure gene regulation. (A) PCR analyses of the *crtB* gene with test‐F1/test‐R1 primers in transformants with spacers of 24, 28, and 30 bp. M, dsDNA size marker; –, the genomic DNA of ΔCR as the negative control. Red triangles indicate transformants with obvious fragment deletions. (B) Whole‐genome sequencing results of transformants with pV10C, pR24R, and pR28R. Red arrows indicate the position of the targeted gene (location on the main chromosome: 2,483,855–2,482,884). The coverage reads of about 50 kb upstream and downstream of the target gene are presented. (C) The growth curves of transformants with different pRSR plasmids. The plasmid pV10C, with mini‐CRISPR containing a nontargeting spacer, served as the control. Error bars indicate the standard deviation (SD) of three independent replicates.

Whether crRNAs with shortened spacers can cause slight cleavage of target DNA on the chromosome and influence cell growth remains to be verified. Herein, we randomly selected 60 ΔCR transformants of *crtB*‐targeting plasmids with spacers of 24, 28, and 30 bp, respectively, for PCR amplification with test‐F1/test‐R1 primers designed from both sides of the *crtB* gene (Figures 4A and S2). Both the 24 and 28 bp transformants generated the PCR products of approximately 2 kb, which were the same size as that of the control, indicating the original length. Importantly, the DNA sequencing results revealed that there was no mutation at the target site in all 60 transformants (Figure S3). However, when the spacer length was 30 bp, 17 transformants had obvious bands between 1 and 2 kb, indicating a fragment deletion in the *crtB* gene (Figures 4A and S2).

Considering that *H. hispanica* is a polyploid haloarchaeon^26^ in which fragment deletions in partial copies of chromosomes can not be detected by the colony PCR assay, whole‐genome sequencing was further carried out for the transformants with a 24 or 28 bp spacer. Unlike colony PCR, whole‐genome sequencing could reflect genome conditions in each cell comprehensively. Transformants with the plasmid pV10C, containing mini‐CRISPR with a nontarget spacer^18^, were used as the control. The plasmid pV10C and *crtB*‐targeting plasmids with a 24 bp spacer or 28 bp spacer were transferred into ΔCR cells. Instead of spreading on the plates, the resuscitating cells were inoculated into the AS‐168SY liquid medium and cultivated until the stationary stage before genomic DNA extraction. Whole‐genome sequencing results revealed that when the spacer length of the *crtB*‐targeting plasmid was 28 bp, the coverage reads of the target gene and nearby genes in *H. hispanica* were significantly reduced, indicating that large fragments might be lost in partial cells or chromosomes (Figure 4B). But notably when the spacer length was 24 bp, the coverage reads of the target gene and nearby genes remained stable (Figure 4B), similar to those of the control, indicating no large fragment deletion. SNP analysis of the samples with 24 and 28 bp spacers revealed no significant point mutations in the target area (Table S3).

To test the impact of spacer length on cell growth, we measured the growth curves of ΔCR transformants with *crtB*‐targeting pRSR plasmids carrying spacers of different lengths (16, 20, 24, 28, and 30 bp), and transformants with the plasmid pV10C were used as the control (Figure 4C). When the length of the spacer was 16, 20, or 24 bp, the growth curve of the transformants was consistent with that of the control, indicating that cell growth was not impaired when targeting the nonessential *crtB* gene. However, transformants with spacer lengths of 28 and 30 bp grew at a lower rate. It suggests that cleavage of the CRISPR‐Cas system may still occur and is in agreement with the results of whole‐genome sequencing, although this effect was insufficient to reduce the transformation efficiency. These results indicate that 24 bp or shorter can be secure spacer lengths for gene repression.

### Selecting spacers of different lengths targeting different locations can regulate gene expression at multilevels

**Figure 5:**
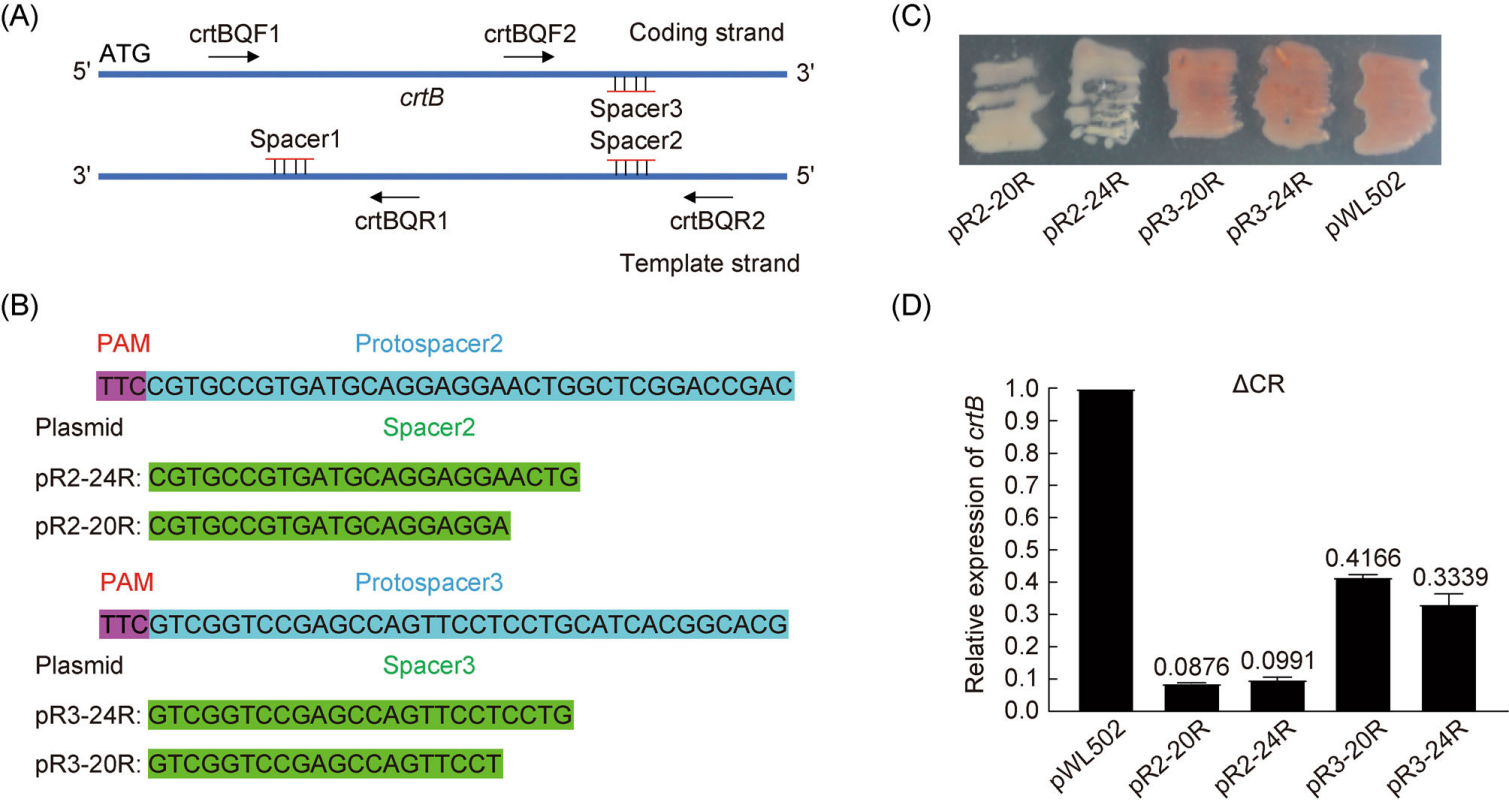
Gene repression effects of CRISPR‐Cas system differ with spacers of different locations. (A) The positions of the targets matched by three spacers on the *crtB* gene. Blue region indicates the coding frame. ATG is the initiation codon. The primers crtBQF1/crtBQR1 and crtBQF2/crtBQR2 were used for a quantitative reverse transcription‐polymerase chain reaction (qRT‐PCR). (B) Schematic of spacer2 and spacer3 with different lengths contained in pRSR plasmids. Magenta background, the PAM sequence; blue background, the protospacer sequence; green background, the spacer sequences. (C) Colonies of transformants with plasmids carrying different spacers of different lengths displayed different colors. (D) Relative expression levels of the *crtB* gene in transformants carrying different pRSR plasmids compared to that with the control plasmid pWL502. Error bars indicate the standard deviation (SD) of three independent replicates.

To regulate gene expression at different levels, we reselected two additional target sites on the *crtB* gene (Figure 5A). The target sites of spacer2 and spacer3 were complementary, located on the template and coding strand of the *crtB* ORF, respectively, and were farther away from the promoter than that of spacer1 (Figure 5A). Two shortened lengths, 20 and 24 bp, were subsequently designed for spacer2 and spacer3, respectively (Figure 5B). Like the results of spacer1, the ΔCR transformation efficiencies of plasmids containing spacer2 or spacer3 were close to that of pWL502 (Figure S4A), indicating a significant loss of interference. As assessed from PCR analyses, there were only original bands for transformants with spacer2 and spacer3 of 24 bp (Figure S4B). Transformants with spacer2 of 20 and 24 bp were white (Figure 5C), indicating that the expression of *crtB* was inhibited strongly. Whereas, transformants with spacer3 were still red (Figure 5C), which showed that the gene repression effect of *crtB* was weak. The relative transcriptional levels of *crtB* in different transformants were measured via qRT‐PCR with the primers crtBQF2/crtBQR2. The transcriptional levels of the *crtB* gene in transformants with pR2‐20R, pR2‐24R, pR3‐20R, and pR3‐24R were reduced to 8.76%, 9.91%, 41.66%, and 33.39% of the control (Figure 5D), respectively. Thus, gene regulation could be achieved at different levels by selecting different target locations with different spacer lengths.

### Type I CRISPR‐Cas system can simultaneously perform gene editing and regulation

According to the above results, when the spacer length was reduced to 24 bp, the interference and primed adaptation disappeared; however, a significant transcriptional inhibition effect could still be observed with an active Cas3 protein. As the type I‐B CRISPR‐Cas system in *H. hispanica* could be harnessed to knock out endogenous genes^26^, the system may be used to simultaneously perform gene editing and repression by altering the spacer length.

**Figure 6:**
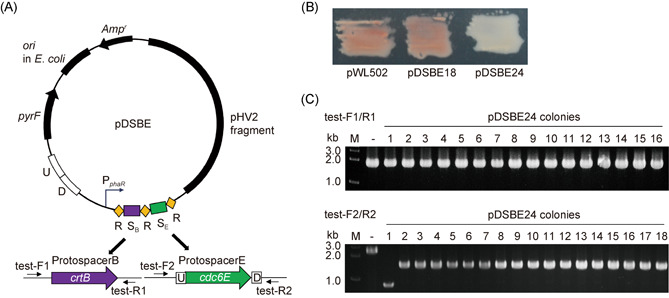
Type I CRISPR‐Cas system is used for simultaneous gene editing and transcriptional repression. (A) Schematic of pDSBE carrying a mini‐CRISPR with two spacers targeting *crtB* and *cdc6E*, and a donor for *cdc6E* deletion. U and D are the upstream and downstream homologous arms of *cdc6E*, respectively. (B) Colonies of transformants carrying pDSBE with different shortened spacers targeting the *crtB* gene, with pWL502 transformant as the control. The pDSBE18 plasmid contains an 18 bp *crtB*‐targeting spacer, and the pDSBE24 plasmid contains a 24 bp *crtB*‐targeting spacer; both plasmids contain a 36 bp *cdc6E*‐targeting spacer. (C) PCR analyses for colonies of pDSBE24 transformants. Primers: test‐F1/test‐R1 for *crtB* and test‐F2/test‐R2 for *cdc6E*. M, dsDNA size marker; –, the genomic DNA of ΔCR as the negative control.

We selected *crtB* and *cdc6E* (HAH_RS09010) as the target genes. Cdc6E participates in replication initiation from one of the multiple origins, and its deletion does not evidently affect cell growth^26^, ^32^. The mini‐CRISPR sequence contained two tandem spacers that targeted these two genes (Figure 6A). For the *crtB* gene, the target site of spacer1 was selected, and spacers of two lengths (18 and 24 bp) were designed to perform gene transcriptional repression. For the *cdc6E* gene, the target site was located in the coding frame and a 36 bp spacer was designed. The DNA donor containing upstream and downstream homologous arms of the *cdc6E* gene was provided in the same plasmid for gene knockout (Figure 6A). Two plasmids, pDSBE18 and pDSBE24, were constructed and named according to the length of the spacer targeting the *crtB* gene, and transferred into ΔCR cells. The color of the pDSBE18 transformants was red, similar to that of the pWL502 transformants, whereas the pDSBE24 transformants were white, indicating that *crtB* transcription was strongly inhibited (Figure 6B).

To determine whether the genes were edited, we randomly selected 60 transformants of pDSBE24 to perform PCR using primers designed from both sides of the genes (Figures 6C and S5). For the *crtB* gene, all 60 colonies had a band of approximately 2 kb, which was the same as that of the control, indicating the original length. For the *cdc6E* gene, 58 colonies had a band of ~1.5 kb, the expected size that was successfully generated using the donor for gene editing (approximately 1.3 kb less than the 2.8 kb band generated in the pWL502 transformants). Notably, the other two colonies had a shorter band of less than 1 kb (Figures 6C and S5). The PCR products of these two colonies were sequenced and aligned with the upstream and downstream sequences of the *cdc6E* gene. The long deletions were found to be caused by micro‐homologous end joining mediated by 20 bp homologous fragments on both sides of the *cdc6E* gene, which was also reported in our previous research^26^.

The above findings demonstrated that the type I CRISPR‐Cas system could be used for simultaneous gene editing and regulation, with both functions being efficient.

## DISCUSSION

The type I CRISPR system is widely distributed in prokaryotes^8^. Interference and primed adaptation are two important processes for the immunity of the CRISPR system and require pre‐existing spacers that fully or partially match the target^2^, ^11^, ^14^, ^18^. The DNA fragments produced by interference may be acquired during primed adaptation^33^ and primed adaptation may defend against mutant phages that would evade the interference^11^. Recently, we have revealed the natural reprogramming of the type I CRISPR effector for gene regulation in which the crRNA‐resembling antitoxin RNA (CreA) served as a guide for the Cascade to inhibit the transcription of the toxin gene (*creT*) by matching an 11 bp seed region^22^. These findings suggest that spacer lengths can be modified to affect functionalities of the CRISPR system, including interference, primed adaptation, and gene regulation. In this study, the spacer length that is required to recognize and bind to the target for transcriptional inhibition is short, while the spacer length required to trigger primed adaptation and interference is longer. Since Cascade effectors can be formed stably with altered stoichiometry^19^, ^20^, ^21^, we speculated that more base pairings were needed to recruit the acquisition machine and Cas3 for acquiring spacers or cleaving the target.

Current gene regulation tools utilizing type I CRISPR‐Cas systems usually involve either knocking out *cas3* gene or mutating its active sites^19^, ^29^, ^30^, ^31^ to prevent the cleavage of target genes. Thus, gene editing and regulation could not be accomplished simultaneously using type I CRISPR‐Cas systems. In this study, we kept the active *cas3* gene and tried to perform both gene regulation and editing. By designing crRNAs with different spacer lengths, we proved that the type I CRISPR‐Cas system with an active *cas3* could be used for gene editing or regulation, even simultaneous gene editing and regulation, which provides a new flexible tool for genetic assay. For example, when we need to target multiple genes, we can inhibit the essential genes with shortened spacers and knock out other genes with full‐length spacers.

As a gene regulation tool, the type I system has the following advantages. First, type I systems have wide ranges of functional spacer lengths^21^, which may help regulate gene transcription to varying degrees. The length of the wild‐type spacer varies mainly from 32 to 39 bp in *H. hispanica* type I‐B CRISPR system^16^. When the spacer was shortened to nearly half of the primary length (only 16 bp), transcription of the target gene could also be reduced to 66.66% of the control (Figure 3D). Moreover, in our previous study, CreA guided Cascade to inhibit CreT transcription mainly by matching the seed region of 11 bp at the promoter^22^. Second, when different target locations were selected in the same gene, different inhibitory intensities were also observed (Figures 3 and 5D). Third, primed adaptation can adapt to various PAM motifs with discriminative adaptation efficiency^25^, which may also work for gene regulation and provide a broad range of targets. Fourth, in type I systems, Cas6 can process pre‐crRNA into mature crRNAs and a single CRISPR array can simultaneously edit or regulate multiple genes. Fifth, by tethering the transcriptional activator or repressor to type I CRISPR‐Cas systems, strong gene regulation can be achieved^34^. Thus, via constructing a spacer library of different target locations and spacer lengths, the type I system can achieve multilevel gene regulation conveniently.

As spacer lengths of different subtypes are sometimes quite different^21^, more experiments are needed to determine the appropriate spacer length range for different type I systems for the application. In this study, when the spacer was shortened to 24 bp, Cas proteins repressed the transcription of *crtB* gene efficiently, while interference and primed adaptation disappeared. On the other hand, in our previous study, when targeting the HHPV‐2 virus with a spacer of 24 bp, very weak primed adaptation could be detected after subculturing three times^18^. We speculated that the intensity of primed adaptation might differ on different targets. By knocking out the leader sequence (like ΔCR strain in this study), Cas1 or Cas2, adaptation can be avoided, while gene editing and regulation will not be affected.

In summary, a systematic study was conducted to elucidate the impact of spacer length on the functionality of the type I CRISPR system (interference, primed adaptation, and gene regulation). By utilizing redesigned crRNAs with spacers of different lengths, we simultaneously conducted gene editing and regulation for the first time with the endogenous type I CRISPR system with active Cas3. The findings of this study may help researchers better understand the flexibility of the type I CRISPR system and provide a novel strategy for delicate manipulation of any genes in a wide range of archaea and bacteria with their endogenous type I CRISPR‐Cas system, especially for those environmental or clinical strains without genetic tools.

## MATERIALS AND METHODS

### Strains and culture conditions

The strains used in this study are listed in Table S1. The *H. hispanica* DF60^27^ strain (a derivative of *H. hispanica* ATCC 33960 with *pyrF* knocked out) and its derivatives were cultured in rich AS‐168 medium (each liter of medium contained 200 g of NaCl, 20 g of MgSO_4_·7H_2_O, 2 g of KCl, 50 mg of FeSO_4_·7H_2_O, and 0.36 mg of MnCl_2_·4H_2_O, 5 g of Bacto casamino acids, 5 g of yeast extract, 3 g of trisodium citrate, and 1.8 g of sodium glutamate; pH 7.2) containing uracil at a final concentration of 50 μg/ml, at 37°C. The strains carrying pWL502^35^ and its derivatives were cultured in AS‐168SY medium (yeast extract‐subtracted AS‐168 medium) to provide selection pressure.

The *Escherichia coli* DH5α strain was used for plasmid construction and cultured in Luria‐Bertani medium (containing 10 g of NaCl, 5 g of yeast extract, and 10 g of tryptone per liter) at 37°C^36^. For the selection of the *E. coli* transformants, ampicillin was added to the final concentration of 100 μg/ml.

### Plasmid construction

The plasmids used or constructed in this study are listed in Table S1, and the primers used for plasmid construction are listed in Table S2. PCR amplification experiments were carried out using high‐fidelity KOD‐Plus DNA polymerase (Toyobo).

The mini‐CRISPR sequence, which comprised the short‐version (constitutive) promoter of the PHA synthesis‐related gene, *phaR* ^37^, and one or two self‐targeting spacers of different lengths flanked by two identical repeats, was synthesized via PCR. The mini‐CRISPR sequence and pWL502 plasmid were digested with the restriction enzymes *Bam*HI and *Kpn*I, and ligated with T4 DNA ligase (New England Lab) to construct a plasmid for gene regulation. For preparing plasmids for gene editing, a DNA donor of 673 bp, containing upstream and downstream homologous arms of the target gene, was cloned into the mini‐CRISPR expression plasmid.

### Plasmid interference assay

After identification via DNA sequencing, the plasmids were transferred into the *H. hispanica* DF60 strain or its derivatives using the polyethylene glycol‐mediated transformation method^38^; the experiment was performed in triplicate. The number of transformants on the plates with different dilution ratios was recorded, and numbers between 30 and 300 were considered valid data. The transformation efficiencies were calculated, and bar charts were generated according to the statistical data.

### Spacer acquisition assay

To monitor spacer acquisition in the DF60 transformants, colonies on the plates with different spacers were lysed with 15 μl of distilled water. Thereafter, 0.5 μl was used as the template for PCR amplification with adaptation primers (Table S2). The PCR conditions were as follows: initial denaturation at 95°C for 5 min; 30 cycles of denaturation at 95°C for 30 s, annealing at 54°C for 30 s, elongation at 72°C for 30 s; and final elongation at 72°C for 10 min. The PCR products were subjected to 1.5% agarose gel electrophoresis.

To monitor spacer acquisition in subcultures, we inoculated transformants with different spacer lengths into 3 ml of AS‐168SY liquid medium and cultured them for 3 days. Serial subcultures were generated at a ratio of 1:15. For each generation, 50 μl of cell culture was centrifuged at 12,000 rpm for 1 min at room temperature, and the supernatant was discarded. The cell sediment was then lysed with 100 μl of distilled water, and 0.5 μl was used as the template for PCR amplification as described above.

### qRT‐PCR

qRT‐PCR was used for quantitative analysis of target gene transcription in different plasmid transformants. Three different colonies of different transformants were selected and inoculated into 10 ml of AS‐168SY medium and cultured to the late logarithmic phase. Thereafter, 3 ml of the culture was used to extract total RNA with TRIzol reagent (Thermo Fisher Scientific). 10 μg of RNA was treated with RQ1 DNase (Promega) to remove genomic DNA, and 1 μg of RNA was used for reverse transcription with M‐MLV reverse transcriptase (Promega) to generate cDNA. qRT‐PCR was carried out using a ViiA^TM^ 7 Real‐Time PCR System (ABI) and the KK4601 SYBR FAST qPCR kit (KAPA Biosystems), with 5 μl of cDNA as a template and 7S RNA gene as an internal control.

### Growth curve plot

For growth curve plotting, the ΔCR transformants with different spacers were inoculated into the AS‐168SY liquid medium. Thereafter, 500 μl of exponential culture was inoculated into 100 ml of fresh AS‐168SY medium and shaken at 200 rpm at 37°C. The OD_600_ of different cultures was measured every 12 h, using an N5000 ultraviolet visible spectrophotometer (Youke), and used to plot the growth curve.

### Whole‐genome sequencing

The plasmid pV10C, also named as pR‐v10‐R^18^, containing mini‐CRISPR with a nontarget spacer, was used as the control. The plasmid pV10C and *crtB*‐targeting plasmids with a 24 bp spacer1 or 28 bp spacer1 were transferred into ΔCR cells as described above with a few modifications as follows: instead of spreading on the plates, after overnight cultivation, the resuscitating cells were inoculated into the AS‐168SY liquid medium and cultivated at 37°C with constant shaking at 200 rpm. The stationary cell culture (800 μl) was centrifuged at 12,000 rpm for 1 min, and genomic DNA was extracted using the phenol–chloroform method. First, the cell pellets were resuspended in 600 μl of double‐distilled water, containing 100 μg/ml proteinase K (Genview), and incubated at 55°C for 30 min. Thereafter, 600 μl of tris‐phenol/chloroform (1:1, v/v) was added and the mixture was incubated at 70°C for 4 h. After centrifugation at 12,000 rpm for 5 min, the supernatant was transferred to a clean 1.5 ml Eppendorf tube. Then, tris‐phenol/chloroform (1:1, v/v) was added at an equal volume and the mixture was centrifuged again; the supernatant was then transferred to a new tube; tris‐phenol/chloroform (1:1, v/v) was repeatedly added until the intermediate layer disappeared. RNaseA (TIANGEN) was added to a final concentration of 50 μg/ml and the solution was incubated at 37°C for 30 min. Thereafter, tris‐phenol/chloroform (1:1, v/v), at an equal volume, was added and the supernatant was transferred to a new tube. Next, chloroform/isoamylol (24:1, v/v), at an equal volume, was added and the supernatant was transferred to a new tube. Then, NaAc (3 M, pH 4.8), in 1/10 volume of the supernatant, and anhydrous ethanol, in twice the volume of the supernatant, were added and the tube was incubated at −70°C for 20 min to precipitate the genomic DNA. Finally, the mixture was centrifuged at 12,000 rpm for 10 min at 4°C, and the sediment was washed with 0.5 ml of 70% ethanol (4°C). The DNA was resuspended in 50 μl of double‐distilled water and used for whole‐genome sequencing by HiSeq. 2 × 150 bp system.

A total of more than 4.3 Gb clean data were generated with each sample at over 1400‐fold sequence depth. The clean reads were mapped to the genome with BWA software^39^. After mapping, the depths at each position were computed using the SAMtools^40^. SNPs frequencies in the target region were identified by counting the frequencies of variant sites relative to the reference sequence based on the alignment files generated from BLASTN.

### Mutation analysis of the target sites via PCR

For transformants of different plasmids, dozens of colonies were randomly scraped from the transformation plates and then lysed with distilled water as a template for PCR amplification using high‐fidelity KOD‐Plus DNA polymerase (Toyobo) and specific primers. For further verification, the PCR products were sequenced.

## AUTHOR CONTRIBUTIONS

Hua Xiang, Luyao Gong, and Ming Li designed and conceived the project. Kaixin Du and Luyao Gong performed the experiments. Kaixin Du, Luyao Gong, Ming Li, Haiying Yu, and Hua Xiang analyzed the data. Kaixin Du and Luyao Gong drafted the manuscript under the guidance of Hua Xiang. All the authors have proofread the manuscript.

## ETHICS STATEMENT

This study has no animal or human experiments. There are no ethical issues involved.

## CONFLICT OF INTERESTS

Hua Xiang, Kaixin Du, Luyao Gong, and Ming Li have filed a related patent.

## DATA AVAILABILITY

All data generated or analyzed during this study are included in this article and its supporting information files.

## Supporting information

Additional Supporting Information may be found online in the supporting information tab for this article.

Supporting information.
