# Supplementary material for: Reprogramming the endogenous type I CRISPR‐Cas system for simultaneous gene regulation and editing in Haloarcula hispanica
Source: mLife. 2022 Mar 17;1(1):40–50. doi: 10.1002/mlf2.12010 (PMC10989794; doi:10.1002/mlf2.12010)
Supplement: Supplementary file 1 — Supporting information. [file MLF2-1-40-s001.docx]

**Table S1** Strains and plasmids used in this study

| **Strains/plasmid** | **Description** | **Source/reference** |
| --- | --- | --- |
| **Strains** | | |
| *E. coli* DH5α | *endA1 phoA supE44 thi-1 recA1 relA1 gyrA96 deoR lacZ*ΔM15Δ(*lacZYA-argF*) *hsdR17* | TsingKe Biotech, Beijing, China |
| *H. hispanica* DF60 | the Δ*pyrF* derivate of *H. hispanica* ATCC 33960 | (1) |
| ΔCR | DF60 with leader and CRISPR array deleted | (2) |
| DC3 | DF60 with *cas3* gene deleted | (3) |
|  |  |  |
| **Plasmids** | | |
| pWL502 | 7.8 kb; an expression vector carrying *pyrF* and its native promoter | (4) |
| pV10C | pWL502 carrying a mini-CRISPR with a spacer targeting virus HHPV-2, also named as pR-v10-R | (5) |
| pR36R | pWL502 carrying a *crtB*-targeting mini- CRISPR with 36 bp spacer1 array, also named as pGK | (6) |
| pR31R | pWL502 carrying a *crtB*-targeting mini-CRISPR with 31 bp spacer1 array | This study |
| pR30R | pWL502 carrying a *crtB*-targeting mini-CRISPR with 30 bp spacer1 array | This study |
| pR28R | pWL502 carrying a *crtB*-targeting mini-CRISPR with 28 bp spacer1 array | This study |
| pR24R | pWL502 carrying a *crtB*-targeting mini-CRISPR with 24 bp spacer1 array | This study |
| pR23R | pWL502 carrying a *crtB*-targeting mini-CRISPR with 23 bp spacer1 array | This study |
| pR22R | pWL502 carrying a *crtB*-targeting mini-CRISPR with 22 bp spacer1 array | This study |
| pR21R | pWL502 carrying a *crtB*-targeting mini-CRISPR with 21 bp spacer1 array | This study |
| pR20R | pWL502 carrying a *crtB*-targeting mini-CRISPR with 20 bp spacer1 array | This study |
| pR16R | pWL502 carrying a *crtB*-targeting mini-CRISPR with 16 bp spacer1 array | This study |
| pR2-24R | pWL502 carrying a *crtB*-targeting mini-CRISPR with 24 bp spacer2 array | This study |
| pR2-20R | pWL502 carrying a *crtB*-targeting mini-CRISPR with 20 bp spacer2 array | This study |
| pR3-24R | pWL502 carrying a *crtB*-targeting mini-CRISPR with 24 bp spacer3 array | This study |
| pR3-20R | pWL502 carrying a *crtB*-targeting mini-CRISPR with 20 bp spacer3 array | This study |
| pDSBE24 | pWL502 carrying a mini-CRISPR, with a 24 bp spacer targeting *crtB* and a spacer targeting *cdc6E*, and a donor for *cdc6E* deletion | This study |
| pDSBE18 | pWL502 carrying a mini-CRISPR, with an 18 bp spacer targeting *crtB* and a spacer targeting *cdc6E*, and a donor for *cdc6E* deletion | This study |

**Table S2** Primers used in this study

| **Primer** | **Sequence (5****′→3′) ^a^** | |
| --- | --- | --- |
| **For construction of mini-CRISPR and donor** | | |
| BamHI-pro-F | | CGCGGATCCCGAAGGGAACATATATGT |
| C1-31bp-R | | CGGGGTACCAAAAAAAAGCTTCAACCCCACGAGGGTTCGTCTGAAACTCGGAGATGTCGTGACGC |
| C1-30bp-R | | CGGGGTACCAAAAAAAAGCTTCAACCCCACGAGGGTTCGTCTGAAACCGGAGATGTCGTGACGCT |
| C1-28bp-R | | CGGGGTACCAAAAAAAAGCTTCAACCCCACGAGGGTTCGTCTGAAACGAGATGTCGTGACGCTCG |
| C1-24bp-R | | CGGGGTACCAAAAAAAAGCTTCAACCCCACGAGGGTTCGTCTGAAACTGTCGTGACGCTCGGCCA |
| C1-23bp-R | | CGGGGTACCAAAAAAAAGCTTCAACCCCACGAGGGTTCGTCTGAAACGTCGTGACGCTCGGCCAG |
| C1-22bp-R | | CGGGGTACCAAAAAAAAGCTTCAACCCCACGAGGGTTCGTCTGAAACTCGTGACGCTCGGCCAGG |
| C1-21bp-R | | CGGGGTACCAAAAAAAAGCTTCAACCCCACGAGGGTTCGTCTGAAACCGTGACGCTCGGCCAGGT |
| C1-20bp-R | | CGGGGTACCAAAAAAAAGCTTCAACCCCACGAGGGTTCGTCTGAAACGTGACGCTCGGCCAGGTC |
| C1-16bp-R | | CGGGGTACCAAAAAAAAGCTTCAACCCCACGAGGGTTCGTCTGAAACCGCTCGGCCAGGTCCT |
| crtB-spacer-BamHI-pro-F | | CGCGGATCCCGAAGGGAACATATATGTTACTGCAGGTACAACACCGAGTTAGGAGATG |
| crtB-S2-pro-R | | CCTGCATCACGGCACGGCTTCAACCCCACGAGGGTTCGTCTGAAACCATCTCCTAACTCGGT |
| crtB-S2-24bp-R | | CGGGGTACCAAAAAAAAGCTTCAACCCCACGAGGGTTCGTCTGAAACCAGTTCCTCCTGCATCACGGCACG |
| crtB-S2-20bp-R | | CGGGGTACCAAAAAAAAGCTTCAACCCCACGAGGGTTCGTCTGAAACTCCTCCTGCATCACGGCACG |
| crtB-S3-pro-R | | ACTGGCTCGGACCGACGCTTCAACCCCACGAGGGTTCGTCTGAAACCATCTCCTAACTCGGT |
| crtB-S3-24bp-R | | CGGGGTACCAAAAAAAAGCTTCAACCCCACGAGGGTTCGTCTGAAACCAGGAGGAACTGGCTCGGACCGAC |
| crtB-S3-20bp-R | | CGGGGTACCAAAAAAAAGCTTCAACCCCACGAGGGTTCGTCTGAAACAGGAACTGGCTCGGACCGAC |
| BamHI-D2-F | | CGCGGATCCTTCGAAACGGAATACCGGGA |
| D2-pro-R | | ACATATATGTTCCCTTCGCTGAAGCACTTGTAGCTTGA |
| CDC6E-R | | CGGGGTACCAAAAAAAAGCTTCAACCCCACGAGGGTTCGTCTGAAACACTGACAGCGTCTTCGAGTTCCTCTAC |
| C1-24bp-R2 | | GTCTTCGAGTTCCTCTACGAGGAGATCGCTTCAACCCCACGAGGGTTCGTCTGAAACTGTCGTGACGCTCGGCCA |
| C1-18bp-R2 | | GTCTTCGAGTTCCTCTACGAGGAGATCGCTTCAACCCCACGAGGGTTCGTCTGAAACGACGCTCGGCCAGGTCCT |
| **For PCR screening and DNA sequencing** | | |
| test-F1 | | GCCCAGACGGGCGACATTCT |
| test-R1 | | GACCATCGCGGTCTGCAAGA |
| test-F2 | | CGTGCGCATTCCGGTCATCT |
| test-R2 | | GGTCGACGAACGTCACTGTA |
| pWL502-seq-F | | TCGACGAACTCTGAACCTATG |
| pWL502-seq-R | | AAGGTCGCTGGGAACTGTAC |
| **For qRT-PCR** | | |
| crtBQF1 | | CGATACAGCAGGAGACCG |
| crtBQR1 | | CATCGCGTCGATGAAGAC |
| crtBQF2 | | TGTACCTCCCACAGGAGA |
| crtBQR2 | | ACCACGTACGGGCGAGCAA |
| 7SF | | TCGATGGTCCGCTGCTCAC |
| 7SR | | GGGGGCGTCCGGTCTGA |
| **For adaptation analysis** | | |
| Leader-F | | CGCGGATCCTCGGTTTCCGTCGAC |
| Spacer1-R | | CGGGGTACCCGGAGGCGATGACTGATG |

^a^ Restriction sites are underlined.

**Table S3** Point mutation analysis by whole-genome sequencing

| **position^a^** | **base^a^** | **pV10C^b^** | **pR24R^b^** | **pR28R^b^** |
| --- | --- | --- | --- | --- |
| -7 | G | 99.76% | 99.74% | 99.78% |
| -6 | C | 99.76% | 99.91% | 99.66% |
| -5 | G | 99.76% | 99.74% | 99.44% |
| -4 | T | 98.56% | 97.94% | 98.54% |
| **-3** | **T** | 98.15% | 98.80% | 98.87% |
| **-2** | **T** | 98.56% | 99.40% | 99.21% |
| **-1** | **C** | 99.68% | 99.57% | 99.44% |
| **1** | **A** | 99.20% | 99.32% | 98.88% |
| **2** | **G** | 99.76% | 99.40% | 99.67% |
| **3** | **G** | 99.44% | 99.74% | 99.56% |
| **4** | **A** | 97.94% | 98.47% | 98.89% |
| **5** | **C** | 99.37% | 99.58% | 99.78% |
| **6** | **C** | 99.76% | 99.75% | 99.55% |
| **7** | **T** | 99.37% | 99.32% | 98.99% |
| **8** | **G** | 99.30% | 99.58% | 99.89% |
| **9** | **G** | 99.53% | 99.74% | 99.44% |
| **10** | **C** | 99.69% | 99.23% | 99.22% |
| **11** | **C** | 99.30% | 99.57% | 99.67% |
| **12** | **G** | 99.53% | 99.83% | 99.67% |
| **13** | **A** | 98.52% | 98.89% | 98.67% |
| **14** | **G** | 99.61% | 99.49% | 99.78% |
| **15** | **C** | 99.54% | 99.83% | 99.67% |
| **16** | **G** | 99.85% | 99.31% | 99.78% |
| **17** | **T** | 98.68% | 98.19% | 99.00% |
| **18** | **C** | 99.38% | 99.48% | 99.56% |
| **19** | **A** | 98.83% | 98.27% | 99.44% |
| **20** | **C** | 99.85% | 99.83% | 99.56% |
| **21** | **G** | 99.61% | 100.00% | 99.66% |
| **22** | **A** | 98.36% | 99.21% | 98.88% |
| **23** | **C** | 99.69% | 99.91% | 99.66% |
| **24** | **A** | 98.60% | 98.86% | 98.55% |
| **25** | **T** | 98.83% | 98.53% | 98.11% |
| **26** | **C** | 99.84% | 99.48% | 99.66% |
| **27** | **T** | 98.43% | 98.01% | 98.33% |
| **28** | **C** | 99.61% | 99.83% | 99.33% |
| **29** | **C** | 99.17% | 99.47% | 99.65% |
| **30** | **G** | 99.58% | 99.82% | 99.88% |
| **31** | **A** | 99.01% | 99.28% | 98.72% |
| **32** | **G** | 99.67% | 99.46% | 99.65% |
| **33** | **G** | 99.35% | 99.82% | 99.88% |
| **34** | **A** | 99.02% | 98.65% | 98.72% |
| **35** | **G** | 99.59% | 99.64% | 99.30% |
| **36** | **A** | 98.11% | 98.38% | 99.54% |
| 37 | C | 99.76% | 99.65% | 99.67% |
| 38 | G | 99.61% | 99.65% | 99.67% |
| 39 | A | 99.22% | 98.94% | 99.34% |
| 40 | T | 98.69% | 98.78% | 99.12% |
| 41 | C | 99.77% | 99.65% | 99.67% |
| 42 | A | 99.54% | 99.48% | 99.56% |
| 43 | A | 99.47% | 99.13% | 99.45% |

^a^ PAM and protospacer1 are corresponding to position -3 to -1 and 1 to 36, respectively, and indicated in bold.

^b^ Percentages show the frequencies of wild type base.

**Fig. S1.** Gene transcriptional repression in the DC3 strain. (A) Relative expression levels of the *crtB* gene in transformants with pRSR plasmids carrying spacers of different lengths compared to that with the control plasmid pWL502. Error bars indicate the standard deviation (SD) of three independent replicates. (B) Colonies of transformants with pRSR plasmids carrying spacers of different lengths displayed different colors.


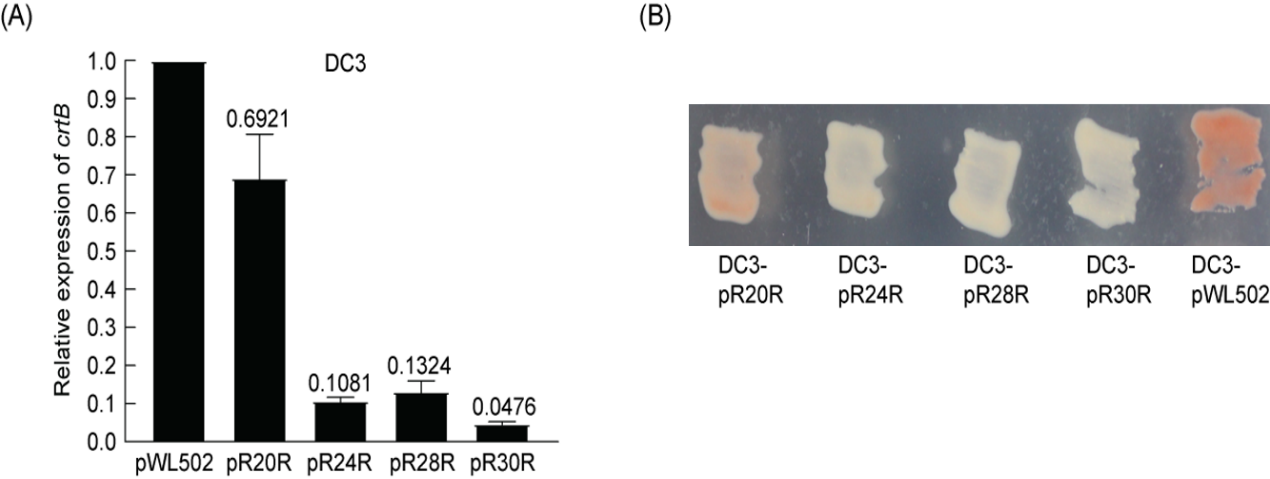


**Fig. S2.** PCR analysis of the *crtB* gene with test-F1/test-R1 primers. Forty-four colonies of ΔCR transformants with pRSR plasmids carrying spacers of 24 bp, 28 bp, and 30 bp size were randomly selected for PCR. M, dsDNA size marker. –, the genomic DNA of ΔCR as the negative control. Red triangles indicate transformants with obvious fragment deletions.


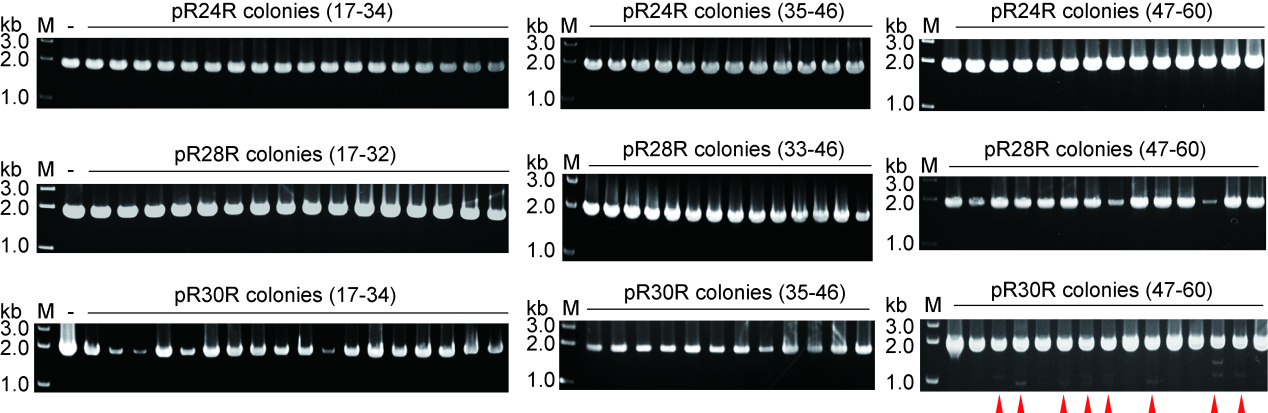


**Fig. S3.** No point mutation in the *crtB* gene protospacer was detected in the ΔCR transformants with pR24R. Sequencing results of ten transformants are presented; sixty transformants were sequenced in total. Red frame sequence indicates PAM. Blue-underlined sequence indicates the protospacer.


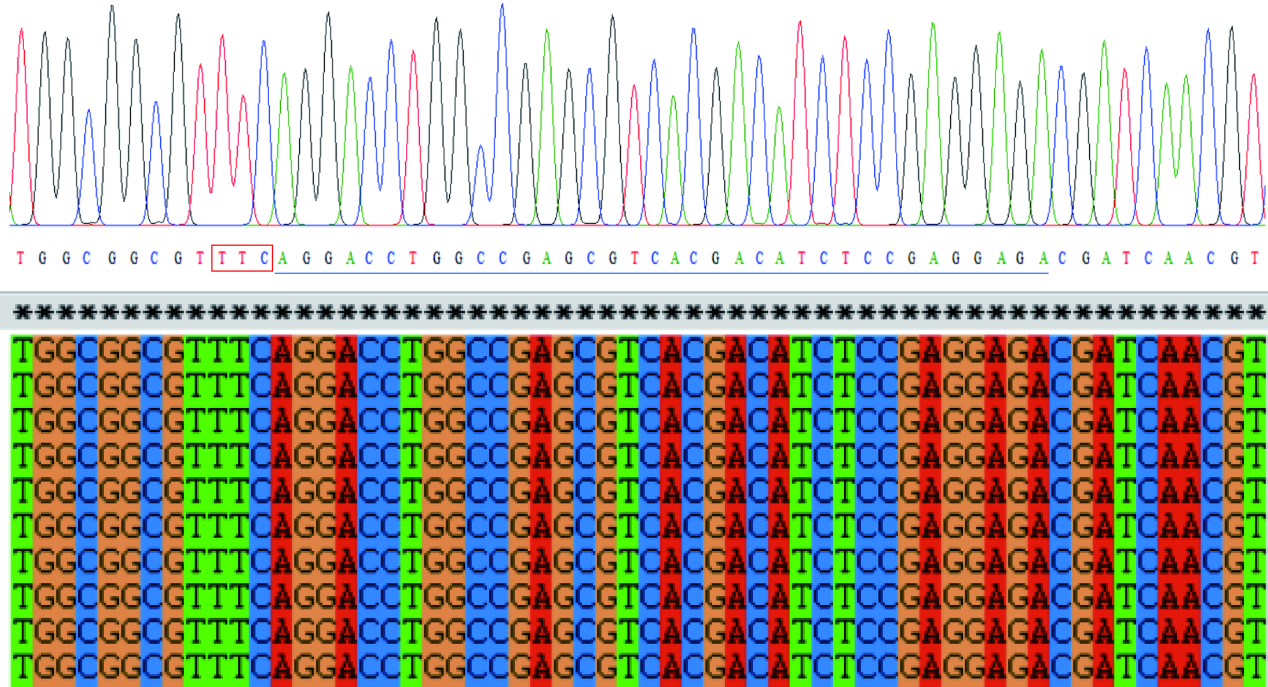


**Fig. S4.** Spacer2 and spacer3 of 24 bp couldn’t cause the cleavage of target DNA in ΔCR. (A) Transformation efficiencies of ΔCR with plasmids carrying spacer2 and spacer3 of 20 bp and 24 bp. Error bars indicate the standard deviation (SD) of three independent replicates. (B) PCR analyses of ten ΔCR transformants with pR2-24R and pR3-24R. M, dsDNA size marker. –, the genomic DNA of ΔCR as the negative control.

**Fig. S5.** PCR analyses of the randomly selected colonies of pDSBE24 transformants. Primers: test-F1/test-R1 for *crtB* and test-F2/test-R2 for *cdc6E*. M, dsDNA size marker. –, the genomic DNA of ΔCR as the negative control.


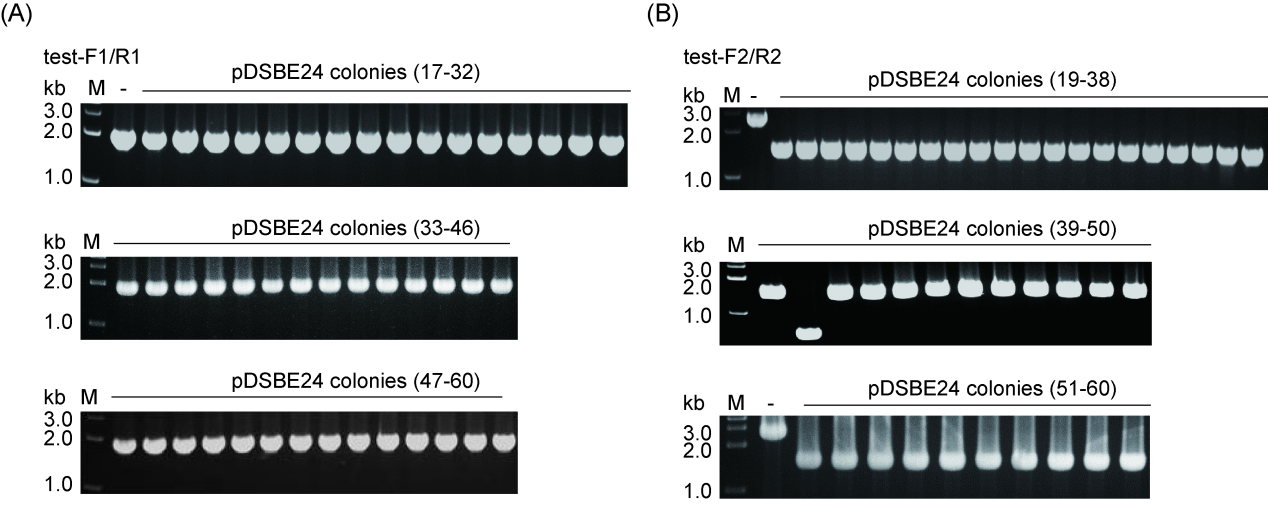


**Supplementary References**

1. Liu H, Han J, Liu X, Zhou J, Xiang H. Development of *pyrF*-based gene knockout systems for genome-wide manipulation of the archaea *Haloferax mediterranei* and *Haloarcula hispanica*. J Genet Genomics. 2011;38:261-9.

2. Wang R, Li M, Gong L, Hu S, Xiang H. DNA motifs determining the accuracy of repeat duplication during CRISPR adaptation in *Haloarcula hispanica*. Nucleic Acids Res. 2016;44:4266-77.

3. Li M, Wang R, Zhao D, Xiang H. Adaptation of the *Haloarcula hispanica* CRISPR-Cas system to a purified virus strictly requires a priming process. Nucleic Acids Res. 2014;42:2483-92.

4. Cai S, Cai L, Liu H, Liu X, Han J, Zhou J, et al. Identification of the haloarchaeal phasin (PhaP) that functions in polyhydroxyalkanoate accumulation and granule formation in *Haloferax mediterranei*. Appl Environ Microbiol. 2012;78:1946-52.

5. Gong L, Li M, Cheng F, Zhao D, Chen Y, Xiang H. Primed adaptation tolerates extensive structural and size variations of the CRISPR RNA guide in *Haloarcula hispanica*. Nucleic Acids Res. 2019;47:5880-91.

6. Cheng F, Gong L, Zhao D, Yang H, Zhou J, Li M, et al. Harnessing the native type I-B CRISPR-Cas for genome editing in a polyploid archaeon. J Genet Genomics. 2017;44:541-8.
